# The effects of knee injury on skeletal muscle function, Na^+^, K^+^-ATPase content, and isoform abundance

**DOI:** 10.14814/phy2.12294

**Published:** 2015-02-12

**Authors:** Ben D Perry, Pazit Levinger, Hayden G Morris, Aaron C Petersen, Andrew P Garnham, Itamar Levinger, Michael J McKenna

**Affiliations:** 1 Institute of Sport, Exercise and Active Living (ISEAL), Victoria UniversityMelbourne, Victoria, Australia; 2 The Park Clinic, St. Vincent's Private HospitalMelbourne, Victoria, Australia; 3 School of Exercise and Nutrition Sciences, Deakin University BurwoodMelbourne, Victoria, Australia

**Keywords:** Disuse, excitability, sodium potassium pump

## Abstract

While training upregulates skeletal muscle Na^+^, K^+^-ATPase (NKA), the effects of knee injury and associated disuse on muscle NKA remain unknown. This was therefore investigated in six healthy young adults with a torn anterior cruciate ligament, (KI; four females, two males; age 25.0 ± 4.9 years; injury duration 15 ± 17 weeks; mean ± SD) and seven age- and BMI-matched asymptomatic controls (CON; five females, two males). Each participant underwent a *vastus lateralis* muscle biopsy, on both legs in KI and one leg in CON. Muscle was analyzed for muscle fiber type and cross-sectional area (CSA), NKA content ([^3^H]ouabain binding), and *α*_1–3_ and *β*_1–2_ isoform abundance. Participants also completed physical activity and knee function questionnaires (KI only); and underwent quadriceps peak isometric strength, thigh CSA and postural sway assessments in both injured and noninjured legs. NKA content was 20.1% lower in the knee-injured leg than the noninjured leg and 22.5% lower than CON. NKA *α*_2_ abundance was 63.0% lower in the knee-injured leg than the noninjured leg, with no differences in other NKA isoforms. Isometric strength and thigh CSA were 21.7% and 7.1% lower in the injured leg than the noninjured leg, respectively. In KI, postural sway did not differ between legs, but for two-legged standing was 43% higher than CON. Hence, muscle NKA content and *α*_2_ abundance were reduced in severe knee injury, which may contribute to impaired muscle function. Restoration of muscle NKA may be important in rehabilitation of muscle function after knee and other lower limb injury.

## Introduction

Severe lower limb injury can substantially reduce lower limb function, leading to physical inactivity (Miyasaka et al. 1991). Almost 3 million people per year in the USA suffer a sports-related lower limb injury, with at least 464,000 being severe enough to require immobilization or unilateral inactivity (Conn et al. 2003). One of the most common debilitating lower limb injuries is anterior cruciate ligament (ACL) tear, and it is estimated that approximately 100,000 people in the USA undergo ACL reconstructive surgery annually, with sporting injuries being the most common cause (Frank and Jackson 1997; Prodromos et al. 2007). Some of the common debilitating effects of lower limb injury include decreased muscle size and strength, decreased joint proprioception, and increased postural sway (Willems et al. 2002; Valderrabano et al. 2006; Lee et al. 2009). Together, these functional decrements adversely impact daily activities and quality of life in patients with lower limb injury (Frobell et al. 2010; Heir et al. 2010).

Muscle disuse due to lower limb injury, such as ACL tear and other knee injuries, can impair both muscle morphology and function, with muscle atrophy and strength loss of the knee extensor muscles common. ACL injury causes a 4.5–13% reduction in cross-sectional area (CSA) in whole muscle and in *vastus lateralis* single muscle fibers, compared to the noninjured leg (Lorentzon et al. 1989; Williams et al. 2005a). This reduction in muscle size in ACL injury is accompanied by an 8–37% lower knee extensor strength (Keays et al. 2001; Makihara et al. 2006). In ACL injury, patients with worse knee function, stability, and “knee related” quality of life also have considerably lower knee extensor CSA and strength (Williams et al. 2005a,b; Roberts et al. 2007). Lower limb injury also causes reduced joint proprioception and increased postural sway, suggesting functional and neuromuscular impairments to balance after injury (Brodie and Sampson 1990; Ditor et al. 2004; Lee et al. 2009).

Part of the weakness and muscle loss after lower limb injury may be related to inactivity, as disuse on both a localized and whole body scale, produces substantial decrements in muscle strength and size (Narici and de Boer 2011). One potential maladaptation in skeletal muscle after injury-induced inactivity is to the Na^+^, K^+^-ATPase (NKA), a protein vital for the maintenance of muscle membrane excitability and muscle contraction (Clausen 2013). The NKA is a heterodimer protein comprising a catalytic *α* and a regulatory *β* subunit (Clausen 2003), each with three isoforms (*α*_1–3_ and *β*_1–3_) detected in human skeletal muscle (Murphy et al. 2004). The *α*_2_ isoform is the dominant NKA *α* isoform in skeletal muscle (Hansen 2001) and has an important role in muscle strength and endurance (Radzyukevich et al. 2013). Mice with a muscle-specific NKA *α*_2_ knockout exhibited substantially reduced exercise performance, impaired in vivo muscle strength and augmented fatiguability (Radzyukevich et al. 2013).

It is well known that the NKA in lower limb muscle is increased with training, as demonstrated by increased vastus lateralis [^3^H]ouabain binding site content (Green et al. 1993, 1999; McKenna et al. 1993; Medbø et al. 2001) and NKA *α*_2_ isoform abundance (Bangsbo et al. 2009; Thomassen et al. 2010). In contrast, the effects of a lower limb injury and associated disuse on skeletal muscle NKA content in humans, and the effects of joint injury on NKA isoform relative abundance remain unknown. Reductions might be anticipated as trunk muscle NKA content was reduced with shoulder injury, being 26% lower in the injured deltoid muscle of patients with shoulder impingement syndrome compared to the noninjured contralateral deltoid muscle (Leivseth and Reikerås 1994).

Elucidating the effects of lower limb injury on muscle NKA content and isoform relative abundance in the lower limbs is important, as these muscles are vital for ambulation, daily functioning and thus quality of life. Furthermore, it is important to also document any reductions in muscle function along with impairments in muscle NKA in injured patients. We therefore investigated the effects of severe knee injury (torn ACL), a common and debilitating lower limb injury, on skeletal muscle NKA content and isoform (*α*_1–3_, *β*_1–2_) relative abundance, knee function, quadriceps muscle strength, muscle fiber CSA, thigh CSA, and postural sway in otherwise healthy, young adult participants compared to both the noninjured leg and healthy-matched controls. It was hypothesized that the knee-injured leg would exhibit reduced muscle NKA content, *α*_2_ isoform abundance, knee extensor strength, thigh and muscle fiber CSA, and increased postural sway compared to both the noninjured leg and to healthy asymptomatic-matched controls.

## Methods

### Participants

Six adults with previously diagnosed knee injury who were scheduled for ACL reconstruction (KI; four females, two males; age: 25.0 ± 4.9 years; body mass: 76.6 ± 5.6 kg; height: 174.2 ± 4.7 cm; BMI: 25.3 ± 2.3 kg·m^−2^ mean ± SD) and seven age- and BMI-matched asymptomatic controls (CON; five females, two males; age: 23.3 ± 2.0 years; mass: 64.0 ± 11.6 kg; height: 170.1 ±  9.1 cm; BMI: 22.0 ± 3.8 kg·m^−2^) gave written informed consent and participated in the study. One participant from the KI group withdrew from the study for personal reasons; hence, analyses were conducted on six KI and seven CON participants. The KI participants were recruited after confirmation that ACL reconstructive surgery was required and had been injured 15 ± 17 weeks prior to surgery (mean ± SD, range 5–50 weeks with an individual injury duration of 5, 6, 7, 9, 15, and 50 weeks). All participants from the KI group damaged their ACL playing recreational sport, and two also had meniscus damage as confirmed by MRI and arthroscopy. No other knee injuries were present. The KI group all played sport at an amateur/recreational level, and none were elite or semielite athletes. Participants were able to ambulate without a walking aid and able to fully extend their knee prior to data collection. Exclusion factors for the KI group included any condition aside from acquired knee injury which would affect physical activity, a BMI over 30 kg·m^−2^, pregnancy, age exceeding 35 years, and any condition contraindicative for muscle biopsies, strength, or balance testing. The control group were matched for age (within 3 years), sex, BMI (within 3 kg·m^−2^), and had to be free from any serious lower limb injury in the past 3 years which required surgery or more than 3 days of immobilization or disuse, in addition to the exclusion criteria already described. Five of the seven control participants were recreationally active reporting to play sport at a local level and/or exercise regularly. The study protocol was approved by the St. Vincent's Hospital Human Research Ethics Committee and the Victoria University Human Research Ethics Committee.

### General design

The knee-injured participants were tested on two separate days. The first day comprised knee extensor muscle strength testing, measurement of one- and two-legged postural sway, thigh anthropometry in both the injured and noninjured legs, as well as completion of a physical activity questionnaire, and a subjective knee function evaluation. Approximately 7 days later, a *vastus lateralis* muscle biopsy was taken from both the injured and noninjured legs while under a general anesthetic, just prior to commencement of their ACL reconstruction surgery. The control participants completed the same functional testing and the physical activity questionnaire, but did not complete the subjective knee function questionnaire. They underwent a *vastus lateralis* muscle biopsy from a single leg, corresponding with the knee-injured participant (right or left leg). In CON, the dominant leg used for thigh CSA, maximal torque, and postural sway measures was defined as the leg the participant used to kick a ball.

### Subjective knee function

Subjective knee function was assessed using the International Knee Documentation Committee (IKDC) Subjective Knee Form, which consists of 18 items relating to injury symptoms, knee function, and sporting physical activity (Hambly and Griva 2010). A greater overall indexed score on the IKDC reflects a higher level of knee functioning and less injury symptoms (Anderson et al. 2006) and is scored by calculating the difference between the raw score and the lowest possible score and then dividing this difference by the range of potential scores, multiplied by 100 (Irrgang et al. 2001). Due to the requirement of healthy knee function for the CON participants, the IKDC was completed only by the KI participants.

### Physical activity questionnaire

The Incidental and Planned Activity Questionnaire (IPAQ) (Hallal and Victora 2004) assessed the physical activity level for all participants. The questionnaire contains 27 questions that estimate the physical activity during the previous week and cover the frequency and duration of occupation-related, transportation, housework/gardening, and leisure-time physical activities. Frequency and duration scores were multiplied to create a total duration for incidental and planned activity, as well as an overall total score summed across all components (h·week^−1^).

### Thigh cross-sectional area

Thigh cross-sectional area (CSA) of each leg was measured using a noninvasive anthropometric method, as described and validated previously (Knapik et al. 1996). In brief, this method involves measurement of thigh circumference at the midpoint of the thigh, which was measured as halfway between the lateral epicondyle and greater trochanter, skin fold assessment, and estimate of femur epicondyle width to calculate an estimate of total thigh CSA in cm^2^. The same experienced investigator performed all measurements. The technique has a standard error of estimate of 10.1 cm^2^ and was highly correlated (*r* = 0.97) with MRI scans of the thigh in a healthy young population (Knapik et al. 1996).

### Muscle fiber cross-sectional area and fiber type

Serial tissue sections of approximately 5 *μ*m thickness were cut at −20°C (HM 550 cryostat, Thermo Fisher Scientific, Scoresby, Vic., Australia) and thaw mounted onto glass microscope slides (Starfrost; ProSciTech, Perth, WA, Australia). Slides were stored at −80°C until analyzed. Tissue sections were removed from −80°C storage and left to air dry for 30 min at room temperature before being fixed in 4% (wt/vol) paraformaldehyde (Sigma-Aldrich, CastleHill, NSW, Australia), permeated with 5% (vol/vol) Triton X-100 (Sigma-Aldrich), and blocked in 3% (wt/vol) bovine serum albumin (Sigma-Aldrich) for 30 min at room temperature in a humid chamber. Sections were rinsed four times with phosphate-buffered saline (PBS) in between each stage. Primary antibodies, diluted in 3% BSA in PBS, were applied and left to incubate in a humid chamber overnight at 4°C. The sections were washed four times in PBS. Secondary antibodies, diluted in 3% BSA in PBS, were applied and incubated for 2 h in a humid chamber at room temperature in the dark. Sections were washed four times and then incubated with the nuclear stain bis-benzimide (Hoechst 33285; Sigma-Aldrich), then mounted in PBS, coverslips applied, and sealed.

Fiber typing of the cross sections was performed using the monoclonal antimyosin antibody A 4.480 specific to Type I myosin (developed by H. Blau and obtained from the Developmental Studies Hybridoma Bank developed under the auspices of the National Institute of Child Health and Human Development and maintained by the University of Iowa, Department of Biological Sciences, IA, USA) and rabbit polyclonal antilaminin antibody was used to delineate the plasma membrane (L9393; Sigma, St. Louis, MO). All unstained fibers were classified as myosin Type II. Secondary antibody-coupled fluorophores used were Alexa Fluor® 488 goat anti-mouse A21042 and Alexa Fluor® 594 donkey anti-rabbit A21207 (Invitrogen, Carlsbad, CA). For negative controls and the assessment of background fluorescence, the primary antibody was omitted and in all cases the fluorescence signal was removed.

Immunostained sections were visualized with an Olympus BX51 fluorescent microscope and digital images were captured using a DP72 color CCD camera and Cell*F software (Olympus Corporation, Tokyo, Japan). Wavelength-specific filters were used to visualize the Alex Fluor 488 and 594 fluorophores, respectively. Captured images were analyzed using ImageJ Software (NIH, Bethesda, MD). Using the laminin-stained membranes for reference, the individual muscle fibers were selected using the polygonal tool and a cross-sectional area was calculated in *μ*m^2^ for each fiber. Only well-cross-sectioned fibers with clear borders were measured, all fibers on the edge of the field of view were also excluded. Muscle fibers counted were 159 ± 77 fibers per sample. Due to small muscle biopsy samples obtained in two KI participants, the muscle fiber CSA is presented for four KI participants and for their four matched controls.

### Peak voluntary strength

Peak voluntary isometric torque was measured as described previously (Levinger et al. 2011; Perry et al. 2013) in both the KI and CON. In brief, a nonextendable strain gauge was attached to the participant's leg at approximately 10 cm above the ankle from a tall chair using a webbing strap with a Velcro fastener. The hip and knee joints were each kept at 90° angle. The distance from the knee joint to the strap around the ankle was measured with a tape measure and used to calculate torque (Nm). The participant exerted maximal force against the strap for 3 sec during each of three trials, with the largest torque recorded and used for analysis.

### Postural sway

Balance was assessed via the anterior–posterior standard deviation (AP-SD) of the center of pressure (Suomi and Koceja 2000; Qiu et al. 2012). The AP-SD was chosen as a measure of postural sway as the muscle groups involved, such as the knee extensors and plantar flexors, are commonly affected by inactivity (Adams et al. 2003; Hackney and Ploutz-Snyder 2011; Narici and de Boer 2011). Postural sway was measured on a portable force platform (400 Series Performance Force Plate; Fitness Technology, Adelaide, SA, Australia) with a data sampling rate of 200 Hz interfaced with a personal computer with software for analysis of postural sway (InnerBalance software, Innervations, Adelaide, SA, Australia). Postural sway AP-SD was measured under quiet conditions during two-legged stance and on each individual leg with both eyes open and closed. The two-legged testing was performed three times, with each trial 30 sec in duration; feet were kept at hip width apart. The one-legged tests were performed four times, each of 10-sec duration. Hands were kept behind the participants back during all conditions and the tests were performed in a quiet environment. The order of one-leg testing was randomly counterbalanced between participants. Data presented are the average of all AP-SD trials for each test.

### Muscle biopsies

For the knee-injured participants, a biopsy was taken from the *vastus lateralis* muscle from both the injured and noninjured legs whilst participants were under a general anesthetic (propofol), immediately prior to the ACL reconstructive surgery. In CON, a local anesthetic was injected into the skin and fascia of the middle third of the *vastus lateralis* muscle (1% Xylocaine). A small incision was made and a muscle sample was taken (∼100–200 mg) using a Bergström biopsy needle. Muscle samples were blotted to remove excess blood, immediately frozen in liquid nitrogen, and stored at −80°C until further analyses. An additional piece of muscle was mounted in OCT (Tissue-Tek; ProSciTech) and frozen in precooled isopentane (Sigma Aldrich, St. Louis, MO) for later immunofluorescent analysis of fiber type and cross-sectional area.

### [^3^H]ouabain binding site content

Analysis of skeletal muscle [^3^H]ouabain binding site content was performed as described previously by Nørgaard et al. (1984) to determine muscle NKA content. In brief, 20 mg of muscle was used; each sample was washed for 2 × 10 min at 37°C in vanadate buffer (250 m·molL^−1^ sucrose, 10 m·molL^−1^ Tris·HCl, 3 m·molL^−1^ MgSO_4_, 1 m·molL^−1^ NaVO_4_; pH 7.3). Muscle samples were then incubated for 2 h at 37°C in vanadate buffer with the addition of [^3^H]ouabain (2.0 Ci.mL^−1^ and 10^−6 ^molL^−1^, PerkinElmer, Boston, MA). The muscle was then placed in ice-cold vanadate solution for 4 × 30 min to remove any unbound [^3^H]ouabain. Muscle samples were blotted on filter paper and weighed before being soaked in 500 *μ*L of 5% trichloroacetic acid and 0.1 m·molL^−1^ ouabain for approximately 20 h. Following this, 2.5 mL of scintillation cocktail (Opti-Fluor; Packard, PerkinElmer) was added before liquid scintillation counting of [^3^H]ouabain. The [^3^H]ouabain binding site content was calculated on the basis of the sample wet weight and specific activity of the incubation buffer and samples and expressed as pmol·g·ww^−1^. The final [^3^H]ouabain binding site content was then calculated as described previously (Nørgaard et al. 1984; Petersen et al. 2005).

### Western blotting: measure of NKA isoform abundance

The NKA isoform relative protein abundance was measured using western blotting. Approximately 10–20 mg of frozen muscle sample was used for NKA immunoblot analyses. Muscle proteins were extracted in ice-cold buffer containing 20 m·molL^−1^ Tris pH 7.8 (Bio-Rad Laboratories, Hercules, CA), 137 m·molL^−1^ NaCl, 2.7 m·molL^−1^ KCl (Merck, Kilsyth, Vic., Australia), 1 m·molL^−1^ MgCl_2_, 5 m·molL^−1^ Na_4_O_7_P_2_, 10 m·molL^−1^ NaF, 1% Triton X-100, 10% Glycerol (Ajax Finechem, Sydney, NSW, Australia), 0.5 m·molL^−1^ Na_4_VO_3_, 1 *μ*g·mL^−1^ Leupeptin, 1 *μ*g·mL^−1^ Aprotinin, 100 m·molL^−1^ PMSF, 1 m·molL^−1^ DTT, and 1 m·molL^−1^ Benzamidine. All reagents were analytical grade (Sigma-Aldrich, St Louis, MO). Samples were homogenized (1:37.5 dilution) for 2 × 20 sec, using a tissue homogenizer (TH220; Omni International, Kennesaw, GA). Homogenates were rotated for 60 min at 4°C and protein concentration of the homogenates was determined using a commercially available kit (DC Protein Assay; Bio-Rad Laboratories).

Muscle NKA isoform analyses did not include any membrane isolation steps to maximize recovery of NKA enzymes (Murphy et al. 2004). Aliquots of the muscle homogenate were mixed with Laemmli sample buffer and proteins were separated with premade 10% sodium dodecyl sulfate–polyacrylamide gel electrophoresis (10%, Criterion TGX, Bio-Rad Laboratories) for 45 min at 200 mA. Following electrophoresis, proteins were transferred to polyvinylidene fluoride membranes (TurboTransfer pack; Bio-Rad) for 7 m at 320 mA using the semi-dry Trans-Blot Turbo Transfer System (Bio-Rad). Membranes were blocked in TBST buffer (10 m·molL^−1^ Tris, 100 m·molL^−1^ NaCl, 0.02% Tween-20) containing 7.5% nonfat milk, for 1 h at room temperature. After being washed (4 × 8 min in TBST), membranes were incubated with the appropriate primary antibody overnight at 4°C. Primary antibodies were diluted in TBS buffer containing 0.1% NaN_3_ and 0.1% albumin bovine serum. All membranes were incubated with the same amount of dilution buffer. Membranes were ponceau stained to confirm complete transfer.

To determine NKA protein abundance, membranes were incubated with antibodies for NKA *α*_1_ (monoclonal *α*6F, developed by D. Fambrough, obtained from the Developmental Studies Hybridoma Bank, maintained by the University of Iowa, USA), NKA *α*_2_ (polyclonal anti-HERED, kindly donated by T. Pressley, Texas Tech University, USA), NKA *α*_3_ (monoclonal, Thermo Scientific, Rockford, IL, # MA3-915), NKA *β*_1_ (monoclonal, Thermo Scientific # MA3-930), NKA *β*_2_ (monoclonal, Becton Dickinson Bioscience, San Jose, Ca, # 610915) using a protocol similar to described elsewhere (Murphy et al. 2004). For the analysis of protein abundance of the *α*_1_, *α*_2_, *β*_1_, and *β*_2_ NKA isoforms, 10 *μ*g of total protein per sample was loaded in each gel, while 15 *μ*g was loaded for the *α*_1_ and *α*_3_ isoform. The *β*_3_ NKA isoform was attempted at several total protein concentrations, but could not be detected. Following incubation with the primary antibodies, membranes were washed in TBST buffer (4 × 8 min) and incubated with the appropriate anti-rabbit (PerkinElmer # NEF812001EA) or anti-mouse (PerkinElmer # NEF822001EA) horseradish peroxidase-conjugated secondary antibodies for 1 h at room temperature. Secondary antibodies were diluted 1:20,000, except *α*_1_ which was diluted 1:5000. After washing the membranes in TBST, immunoreactive proteins were detected using chemiluminescence reagents (Bio-Rad) and quantified by densitometric scanning (VersaDocTM Imaging System; Bio-Rad). The abundance of NKA isoforms was normalized to total protein content of the lane (blot density divided by lane protein content, multiplied by 100) as assessed using a coomassie stain (50% methanol, 50% H_2_O and 0.1% Brilliant Blue R-250 powder) on the membrane (Welinder and Ekblad 2011). All samples, including controls, were run on the same gel.

### Statistical analyses

All data are reported as mean ± standard deviation (SD). Statistical significance was accepted at *P* < 0.05. Log transformation was performed on the muscle cross-sectional area data due to the data not initially being normally distributed. Paired *t*-tests were used to determine differences between the injured and noninjured legs in the KI participants for muscle fiber CSA, [^3^H]ouabain binding site content, NKA isoform abundance; independent t-tests were performed to compare the above measures to the CON group. A two-way mixed model ANOVA (leg, group) was used to assess the difference in knee extensor strength, thigh cross-sectional area, and postural sway between the injured and noninjured legs of the KI group and compared to CON. Correlations between [^3^H]ouabain binding site content, NKA *α*_2_ isoform relative abundance, strength, muscle CSA, subjective knee function, and duration of injury were analyzed using Pearson's product-moment correlation coefficient. Magnitudes of change using Cohen's effect size (ES) was assessed on all variables except physical activity and measures between the dominant and nondominant legs in the CON; ES was defined as small, 0.2–0.6; moderate, 0.6–1.2; large, 1.2–2.0; and very large, 2.0–4.0 (Batterham and Hopkins 2006; Hopkins 2007). Effects with less certainty (magnitude of <75%) were classified as no meaningful difference (Batterham and Hopkins 2006; Hopkins 2007) and were not reported. T-tests and mixed models ANOVA were calculated using SPSS version 20 (SPSS Inc., Champaign, IL) and effect size calculated via a custom spreadsheet (Hopkins 2007).

## Results

### Subjective knee function and physical activity

In KI, subjective knee function was 53.8 ± 18.7 a.u. (arbitrary units, range 37.9–85 a.u.), which places knee function and symptoms with daily activity in the lowest 5–15th percentile of normative data from the 18- to 34-year-old age group (Anderson et al. 2006). Total physical activity time did not differ between KI and CON (10.7 ± 7.0 vs. 12.1 ± 4.73 h·week^−1^, respectively, *P* = 0.65), nor did leisure/planned activity time (KI: 2.2 ± 2.3 vs. CON: 3.7 ± 2.7 h·week^−1^, *P* = 0.29).

### Skeletal muscle NKA content

**Figure 1:**
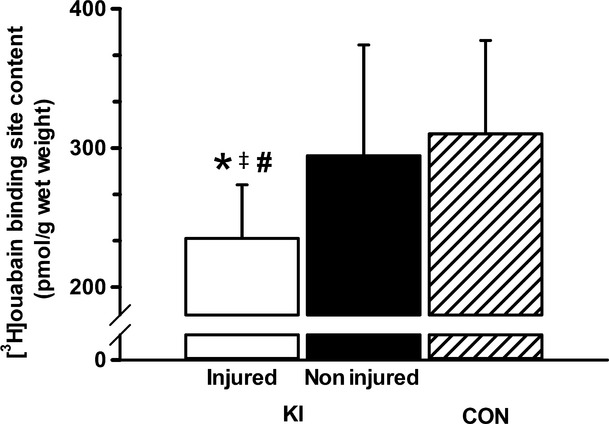
*Vastus lateralis* muscle [^3^H]ouabain binding site content in the injured and noninjured leg of KI and in CON. *Less than the noninjured leg (*P* < 0.05), ^#^Less than CON (*P* < 0.05), ^&ddagger;^Moderate effect size compared to noninjured leg. Values are Mean ± SD, KI, *n* = 6 for each leg; CON, *n* = 7.

The skeletal muscle [^3^H]ouabain binding site content in the knee-injured leg was 20.2% lower than in the noninjured leg (*P* = 0.045, ES: 0.8 ± 0.61) and 22.5% lower than in CON (*P* = 0.043, ES: 1.19 ± 0.91, Fig.1).

### Skeletal muscle NKA isoform abundance

**Figure 2:**
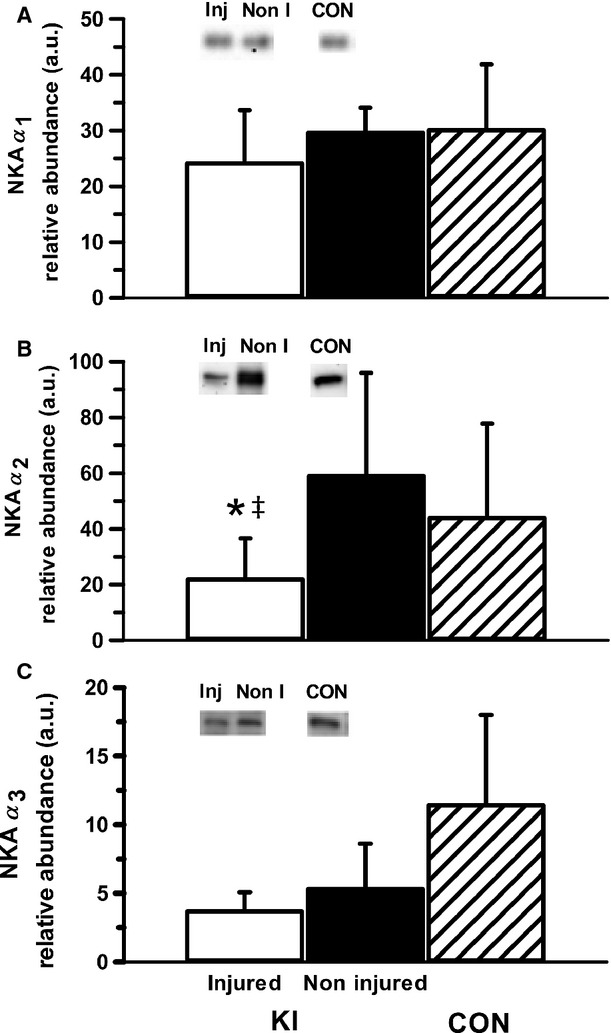
Muscle NKA *α*_1_ (A), *α*_2_ (B), and *α*_3_ (C) isoform relative abundance from the *vastus lateralis* of the injured and noninjured legs of participants with knee injury (KI) and from a single leg of age- and BMI-matched controls (CON). Representative western blots are included; “Inj” represents the knee-injured leg, “Non-I” represents the noninjured leg, and “CON” represents the control group. Unfilled bars represent the injured leg from KI, filled bars represent the noninjured leg from KI and the hatched bars represent CON. *Less than noninjured leg (*P* < 0.05), ^&ddagger;^Moderate effect size compared to noninjured leg. Values are Mean ± SD, *n* = 6 for each leg KI, *n* = 7 CON.

The NKA *α*_2_ abundance in the injured leg was 63% lower than the noninjured leg in KI (*P* = 0.032, ES: 1.11 ± 0.76, Fig.2), but did not differ significantly from CON, although a moderate effect size was found (*P* = 0.167, ES: 0.77 ± 0.91). There were no differences in NKA *α*_1_ or *α*_3_ between the injured and noninjured legs in the KI group, or between KI and CON (Fig.2).

**Figure 3:**
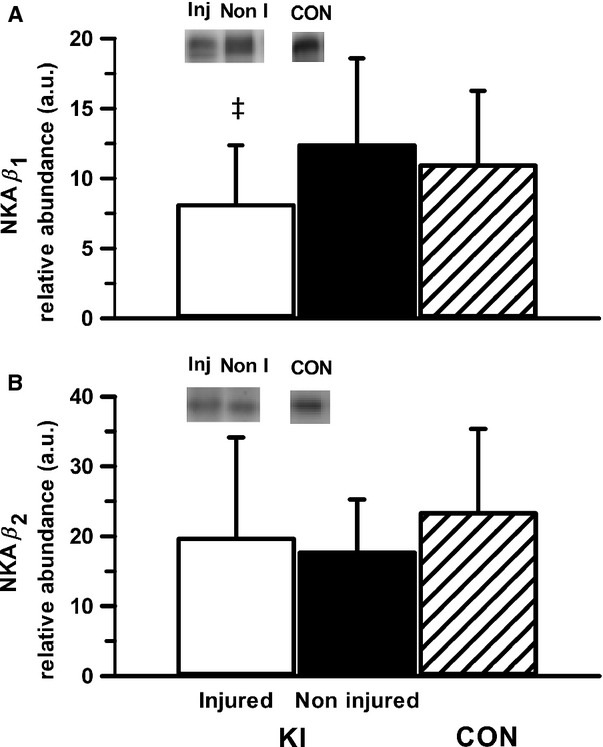
Muscle NKA *β*_1_ (A) and *β*_2_ (B) isoform relative abundance from the *vastus lateralis* of the injured and noninjured legs of participants with knee injury (KI) and from a single leg of age- and BMI-matched controls (CON). Representative western blots are included; “Inj” represents the knee-injured leg, “Non-I” represents the noninjured leg, and “CON “represents the control group. Hollow bars represent the injured leg from KI, filled bars represent the noninjured leg from KI and the hatched bars represent the CON. ^&ddagger;^Moderate effect size compared to noninjured leg in KI. Values are Mean ± SD, *n* = 6 for each leg KI, *n* = 7 CON.

**Figure 4:**
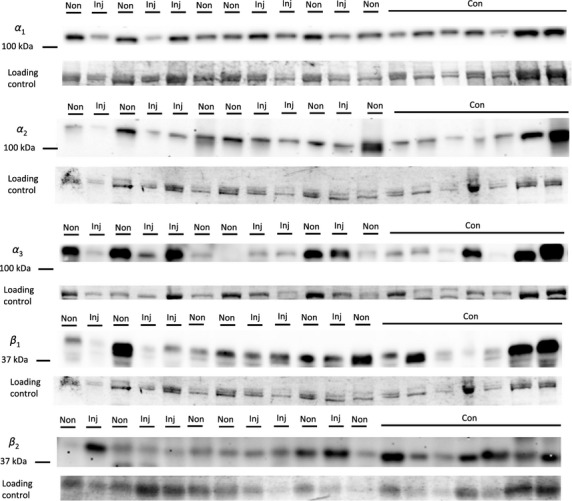
Representative western blot images for each NKA isoform analyzed (*α*_1-3_ and *β*_1-2_), including example of the coomassie stain used to normalize blot density to the total protein content of the sample. “Inj” represents the injured leg from the KI group, “Non” represents the noninjured leg from the KI group, and “CON” represents the control group.

While there was no significant difference in *β*_1_ abundance within the KI group or compared to CON, there was a moderate effect size in *β*_1_ abundance between the injured and noninjured leg in KI (34.5%, *P* = 0.17, ES: 0.68 ± 0.87; Fig.3). There were no differences or meaningful effect sizes in the relative abundance of the *β*_2_ isoform either between the injured and noninjured legs, or in the knee-injured leg compared to the CON group (Fig.3). Representative western blots are shown in Fig. 4.

### Muscle **fiber** cross-sectional area

**Figure 5:**
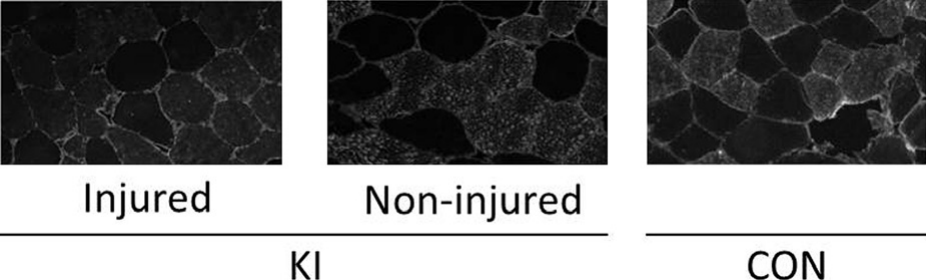
Representative immunofluorescence images in grayscale from the injured and noninjured leg of the KI group, and from the CON group.

In KI, there were no significant differences in *vastus lateralis* type I, type II, or combined muscle fiber CSA between the injured and noninjured leg (Table1), or between CON group and the injured leg in KI (*n* = 4, *P* > 0.22). In addition, there was no difference in the percentage of type I muscle fiber distribution between legs and groups (*P* > 0.21, Table1). There was a small effect size for the combined muscle fiber CSA to be lower in the injured than in the noninjured legs in KI (−21.6%, *P* = 0.33, ES: 0.41 ± 0.35) (Fig 5).

**Table 1:** Muscle fiber cross-sectional area and fiber-type distribution of the *vastus lateralis* in patients with severe knee injury (KI) and in healthy, matched controls (CON).

Group	KI		CON
Leg	Injured	Noninjured	
Muscle fiber CSA (μm^2^)			
Type I	3719.7 ± 1746.7	4649.4 ± 768.9	4399.7 ± 1946.1
Type II	3752.9 ± 2693.5	4881.3 ± 1376.1	4468.9 ± 1888.1
Combined	3736.2 ± 2161.3*	4765.1 ± 992.8	5154.6 ± 1538.7
Type I fiber distribution (%)	56.8 ± 12.1	42.0 ± 28.4	49.2 ± 13

* Small effect size.

Values are Mean ± SD. *n* = 4 KI, *n* = 4 CON. Combined denotes both type I and II fibers pooled.

### Thigh cross-sectional area and peak knee extensor torque

Total thigh CSA in the injured leg was 7.1% lower than the noninjured leg in KI (*P* = 0.021, ES: 0.61 ± 0.38, Table2). The KI thigh CSA was higher than in CON (*P* = 0.039). In KI, the peak isometric knee extensor torque was 21.2% lower in the injured compared to the noninjured leg (*P* = 0.021, ES: 0.59 ± 0.38, Table2). Knee extensor isometric torque expressed relative to body mass was similarly 21.7% lower in the injured than the noninjured leg (*P* = 0.027, ES: 0.7 ± 0.46). Peak knee extensor torque relative to thigh CSA also tended to be lower in the injured compared to the noninjured leg (−15.5%, *P* = 0.086, ES: 0.41 ± 0.35, Table2). There were, however, no differences between the dominant and nondominant legs in CON, or between groups (KI vs. CON) in any of the peak torque measures (*P* > 0.28).

**Table 2:** Thigh cross-sectional area (CSA) and maximal isometric torque expressed in absolute units, relative to body mass and to thigh CSA.

Group	KI		CON	
Leg	Injured	Noninjured	Injured	Noninjured
Thigh CSA (cm)	150.5 ± 16.2*^,^**	162.0 ± 15.3	127.1 ± 29.3	125.3 ± 26.9
Peak torque (Nm)	94.6 ± 35.1*^,^**	120.2 ± 37.8	106.4 ± 28.4	102.7 ± 23.5
Peak torque relative to body mass (Nm·kg^−1^)	1.22 ± 0.39*^,^**	1.56 ± 0.42	1.61 ± 0.26	1.56 ± 0.17
Peak torque relative to CSA (Nm·cm^−2^)	0.63 ± 0.24**	0.75 ± 0.23	0.84 ± 0.14	0.82 ± 0.09

* Less than to noninjured leg (*P* < 0.05; interaction effect between leg and group).

** Small effect size compared to noninjured leg.

Mean ± SD. *n* = 6 for KI, *n* = 7 for CON.

### Postural sway

There was a 43% greater two-legged AP-SD with eyes closed in KI compared to CON (*P* = 0.04, ES: 1.16 ± 0.96), but no difference with eyes open (*P* = 0.14, ES: 0.74 ± 1.0, Table3). There were no differences in single-leg AP-SD between the injured and noninjured legs in KI with either the eyes open (*P* = 0.75, ES: 0.08 ± 0.97) or eyes closed conditions (*P* = 0.13, ES: 0.46 ± 0.84, Table3). There was no difference in postural sway between legs in CON in either condition (*P* > 0.33), or any overall difference between groups in either the eyes open or closed conditions, although there was a trend for higher overall single-leg postural sway with eyes open in KI compared to CON (*P* = 0.06).

**Table 3:** Two- and one-legged postural sway (AP-SD) in KI and CON groups with eyes open and eyes closed.

	Eyes open	Eyes closed
Two-legged AP-SD (cm)		
KI	0.31 ± 0.18	0.30 ± 0.09*,**
CON	0.19 ± 0.05	0.21 ± 0.05
One-legged AP-SD (cm)		
KI		
Noninjured	0.59 ± 0.09	1.12 ± 0.19
Injured	0.65 ± 0.16	1.26 ± 0.26
CON		
Dominant	0.51 ± 0.13	1.02 ± 0.09
Nondominant	0.49 ± 0.20	1.19 ± 0.45

* Higher than CON, *P* < 0.05.

** Moderate effect size compared to CON.

Values are Mean ± SD. *n* = 6 for KI, *n* = 7 for CON.

### Correlations

**Figure 6:**
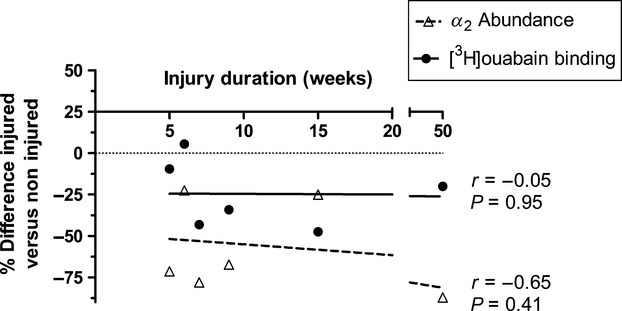
Scatterplot representing muscle [^3^H]ouabain binding site content and NKA *α*_2_ abundance compared to knee injury duration (weeks) in the injured leg from the KI group. Solid regression line represents [^3^H]ouabain binding site content (p.mol per gram wet weight^−1^), and the broken regressions line represents NKA *α*_2_ abundance (A.U.). *n* = 6 for KI.

There were no significant correlations between any of muscle [^3^H]ouabain binding site content (Fig.6), NKA *α*_2_ abundance (Fig.6), strength, thigh CSA, IPAQ, IKDC scores, and duration of injury in either pooled data or within individual groups (*P* > 0.05).

## Discussion

This is the first study to demonstrate that lower limb injury is associated with lower skeletal muscle NKA content and *α*_2_ relative abundance; these changes were also coincident with lower thigh muscle CSA and impaired muscle function, evidenced via reduced peak muscular strength. These skeletal muscle maladaptations with lower limb injury are consistent with inactivity-induced changes and raise the possibility of impaired muscle excitability and contractility in these patients.

We report that severe lower limb injury, in the form of an ACL tear, lowered skeletal muscle NKA content, as measured by [^3^H]ouabain binding site content. A decline in NKA content is important as this may impair myocytic Na^+^/K^+^ exchange and membrane potential during exercise, which may ultimately contribute to reduced muscle function via inexcitability and fatigue (Clausen 2003; McKenna et al. 2008). Whether the injury is restricted to the upper or lower limbs, as well as the time-course and severity of the injury might each contribute to the lower muscle NKA content. The 20% lower [^3^H]ouabain binding site content in the injured leg compared to the noninjured leg in this study is remarkable given that a similar lowering of NKA content (26%) was found between the injured and noninjured deltoid muscles of patients with shoulder impingement syndrome, despite a longer minimum injury duration of 8 months (Leivseth and Reikerås 1994). It remains unclear whether this difference is due to the muscle sampled or the nature of the injury. In addition to these findings being the first in lower limb injury, our work also further extends these earlier findings from Leivseth and Reikerås (1994) through detailed analysis of NKA isoforms, as well as inclusion of a control group and measures of muscle strength.

A second key finding was the 63% lower muscle NKA *α*_2_ isoform protein abundance found in the injured leg compared to the noninjured leg in KI; this is two-fold greater than the reduction in [^3^H]ouabain binding site content. This discrepancy between the [^3^H]ouabain binding site content and *α*_2_ is surprising considering that ∼75–80% of NKA *α* isoforms is the *α*_2_ isoform, at least in rat EDL muscle (Hansen 2001). A similar proportion might be anticipated in human muscle, but given the different fiber-type composition in human *vastus lateralis* compared rat EDL, this is not known. This disparity between the muscle [^3^H]ouabain binding site content and *α*_2_ findings may reflect the semiquantitative, nonmolar and relative nature of western blotting analysis. The lower NKA *α*_2_ abundance in the knee-injured leg indicates that the decline in muscle NKA content after injury is likely to be primarily due to a reduction in the abundance of *α*_2_ isoform. This has important adverse implications for muscle function and fatiguability. Muscle-specific NKA *α*_2_ knockout mice exhibited vastly reduced electrically stimulated muscle strength and increased in vivo muscle fatigability*,* and a substantial reduction in performance on a graded treadmill test (Radzyukevich et al. 2013). This also occurred despite compensatory upregulation of the NKA *α*_1_ isoform, indicating the specific importance of NKA *α*_2_ for skeletal muscle function (Radzyukevich et al. 2013). In addition, studies utilizing rat in vitro skeletal muscle preparations have demonstrated that incubation in 10^−6^ molL^−1^ ouabain, which inhibits a significant fraction of NKA *α*_2_ substantially impaired muscle force and recovery (Clausen 2013). In one study incubation in 10^−6^ molL^−1^ ouabain caused a 28% inhibition of NKA *α*_2_ abundance and 22% decrease in the time to inhibition of muscle force, with a concomitant reduction in NKA activity, as measured via ^86^Rb uptake (Clausen and Everts 1991). Hence, even a small decrease in NKA *α*_2_ relative abundance or [^3^H]ouabain binding site content, as seen in this study with severe knee injury, could potentially impair muscle function and contractility. There were no differences with knee injury to the other NKA isoforms (*α*_1_, *α*_3_, *β*_1_, or *β*_2_) in skeletal muscle, which raises the intriguing question about why other *α* isoforms were also not reduced with knee injury. Furthermore, despite the difference in [^3^H]ouabain binding site content, there was no difference in NKA *α*_2_ between the control and the injured leg. While controls were matched for BMI and age, matching physical activity history between an injured population and a control group was difficult. Combined with the semiquantitative nature of western blotting these two factors may have contributed to the lack of difference in NKA *α*_2_ abundance between the injured leg and controls. Other limitations of this study were the lack NKA activity measurement or any measurement of systemic ion homeostasis, such as venous or arterial [K^+^] during fatiguing exercise. However, measurement of both NKA activity and plasma [K^+^] would be most valuable before and after intense, fatiguing exercise. This was unfortunately not safely possible in this injured population. An inevitable weakness of recruiting participants with severe knee injury, especially in a small sample, is the high variability of injury severity and duration between participants. The duration of injury in this study ranged from 5 to 50 weeks; hence, it is difficult to accurately assess at what duration of injury muscle NKA decreases. Investigation into the associations between muscle strength, excitability, fatigability, and NKA content in a larger pool of patients after lower limb injury is required. Future research following lower limb injured patients after improvement of knee function by either surgery or rehabilitation is important to investigate the reversibility and time-course of the reduced skeletal muscle NKA content. In particular, investigation of the role of central limitation and other neural maladaptations after ACL injury (Mizner et al. 2005) and its association with muscle NKA after both injury and rehabilitation will provide useful information as to whether central limitation is associated with changes in muscle NKA, or whether changes in muscle NKA simply reflect disuse. If muscle NKA is only associated only with disuse, therapies which promote even gentle muscle activity may reduce the decline of muscle NKA content after severe knee injury.

The reduction in muscle NKA content in this study was substantially less than the 34–58% lower NKA content reported after complete cervical spinal injury (Ditor et al. 2004; Boon et al. 2012), although the decrease in NKA *α*_2_ isoform abundance in this study was similar to the 50% decrease seen after 12 months of spinal injury (Boon et al. 2012). Spinal injury causes considerable dysfunction to muscle excitability and disruption of neurotrophic factors within skeletal muscle independent of disuse (Shields 2002). Hence, a greater apparent decline in skeletal muscle [^3^H]ouabain binding site content after spinal injury likely reflects the combined maladaptive effects of spinal injury and muscle disuse. However, the Boon et al. (2012) study also investigated patients with partial spinal injury whom maintained sufficient neuromuscular function to ambulate. Despite these patients likely having some degree of neural damage, there was no change in muscle NKA *α*_1_ and *α*_2_ isoform abundance 3–12 months after injury, suggesting even the limited physical activity these patients were capable of maintaining muscle NKA isoform abundance. The reduction in both NKA content and *α*_2_ isoform abundance in only the injured leg in our study suggests that the injury caused substantial localized disuse which was not apparent in the noninjured leg. As the skeletal muscle innervation is not directly impaired by ACL tear and other knee injuries, the 20% reduction in [^3^H]ouabain binding site content found here likely represents the direct effects of disuse per se on the injured leg rather than the knee injury. However, the effects of reduced central activation (Mizner et al. 2005) and other neural maladaptations (Madhavan and Shields 2011) after ACL tear and other knee injuries on muscle NKA content are not known. Hence, the neural maladaptations caused by knee injury, which were not assessed in this study, may have also contributed to the lower muscle NKA content in the injured leg. Interestingly, the magnitude of this difference (20%) is proportionally similar but opposite of the increase in NKA content found after training (Green et al. 1993, 1999; McKenna et al. 1993; Medbø et al. 2001). This strongly suggests that the downregulation of NKA content is an effect of physical inactivity per se.

The underlying mechanism causing reduced NKA *α*_2_ abundance and NKA content in skeletal muscle with disuse is not fully understood. Transient chronic increases in muscle intracellular [Na^+^] increase NKA content and *α*_2_ abundance in cultured rat muscle (Brodie and Sampson 1990) and this is suspected, yet untested, to be the mechanism which causes increased NKA content with training (Clausen 2003). Hence, one potential mechanism behind the decrease in NKA content after knee injury is also a disturbance in intracellular Na^+^ regulation. Three weeks of hind-limb unloading in rats caused an increase in Na^+^ channel density (Desaphy et al. 2001) and corresponded to a change in muscle phenotype; but whether this occurs with knee injury and with reduced muscle NKA is not clear. Another speculative pathway of NKA downregulation is through the biochemical signaling pathways activated by inactivity in muscle; such as atrophy-inducing proteins and pathways such as FoxO, Atrogin-1 and MuRF-1 (Bodine et al. 2001). Further research is required to elucidate the cellular mechanistic causes of decreased NKA content after disuse and injury.

The impairment in knee extensor peak strength (−21%) and thigh muscle CSA (−7.1%) in this study is typical of patients with ACL tear, although other studies have reported strength loss as high as 38% and with up to a 14% reduction in CSA of the knee extensors (Lorentzon et al. 1989; Williams et al. 2005a; Makihara et al. 2006). The average IKDC score of 53.8, the lowest ∼5–15% of knee function for their age group (Anderson et al. 2006) suggests that all participants had some level of knee dysfunction due to knee injury that adversely impacted daily and leisure activities with the injured leg. However, there were no significant differences in leisure and overall physical activity time between KI and CON. Due to the role of physical activity in regulating muscle NKA content, the potential mismatch of physical activity levels between the KI and CON groups is a limitation of our study. Analysis of preinjury physical activity levels of patients may provide a greater insight into the effects of knee injury on physical activity and should be utilized in future research, as the CON group may not have been as active as the KI group prior to injury.

A reduction in weight bearing on the knee-injured leg, commonly seen in patients following ACL injury and other severe lower limb injuries, likely mediated knee extensor atrophy. Only 5 days of immobilization was sufficient to cause a 3.5% decrease in quadriceps muscle CSA in humans (Wall et al. 2014), with more substantial decreases in CSA (8–10%) occurring after 2–3 weeks (Narici and de Boer 2011). However, the strength loss measured here likely reflected a combination of knee dysfunction and disuse-mediated maladaptations independent of atrophy. This was evident as when normalized for muscle CSA, knee extensor torque still tended (*P* = 0.086) to be 15% lower in the injured than in the noninjured leg. We also cannot exclude the possibility that patients produced less effort due to pain or fear of producing further damage, which was not recorded during the testing. Regardless, this reduction of muscle function is of clinical importance; ACL injured patients with lesser atrophy and strength loss have better knee function (Williams et al. 2005b). Hence, the disuse induced by knee injury is likely to at least partially mediate the impairment of muscle function and reduction in CSA in the several weeks after injury, which may then further contribute to reduced joint function (Williams et al. 2005a).

Despite the lower knee and muscle function after knee injury, indicating the substantial adverse impact of the injury, there was no significant impairment in postural sway between the injured and noninjured legs in the KI group. However, there was a greater two-legged postural sway in KI compared to the CON. The lack of change in single leg postural sway contrasts previous research investigating single-leg postural sway after ACL injury (Ageberg et al. 2005; Lee et al. 2009). This may be because this study used a different postural sway methodology to both previous studies, had a smaller sample size and/or used a population with more recent ACL injury. The higher postural sway only in two-legged stance with eyes closed in KI compared to CON suggests some impairment in balance that was not specific to the injured leg, perhaps due to impaired knee proprioception (Barrack et al. 1989; MacDonald et al. 1996; Roberts et al. 2007; Lee et al. 2009), although it is unusual that increased postural sway was not detected in the injured leg alone if this was the case. Hence, further investigation is required to better understand the impairment of postural sway after knee injury and importantly its role in limb and muscle function.

## Conclusions

In conclusion, lower limb injury was associated with reductions in skeletal muscle NKA content and *α*_2_ isoform abundance, and was accompanied by functional impairments in knee extensor strength and reduced thigh muscle cross-sectional area. The reduction in skeletal muscle NKA content may contribute to the impairment of muscle function after serious knee injury.

## References

[b1] Adams GR, Caiozzo VJ, Baldwin KM (2003). Skeletal muscle unweighting: spaceflight and ground-based models. J. Appl. Physiol.

[b2] Ageberg E, Roberts D, Holmström E, Fridén T (2005). Balance in single-limb stance in patients with anterior cruciate ligament injury relation to knee laxity, proprioception, muscle strength, and subjective function. Am. J. Sports Med.

[b3] Anderson AF, Irrgang JJ, Kocher MS, Mann BJ, Harrast JJ (2006). The International Knee Documentation Committee Subjective Knee Evaluation Form Normative Data. Am. J. Sports Med.

[b4] Bangsbo J, Gunnarsson TP, Wendell J, Nybo L, Thomassen M (2009). Reduced volume and increased training intensity elevate muscle Na^+^-K^+^ pump *α*_2_-subunit expression as well as short-and long-term work capacity in humans. J. Appl. Physiol.

[b5] Barrack RL, Skinner HB, Buckley SL (1989). Proprioception in the anterior cruciate deficient knee. Am. J. Sports Med.

[b6] Batterham AM, Hopkins WG (2006). Making meaningful inferences about magnitudes. Int. J. Sports Physiol. Perform.

[b7] Bodine SC, Latres E, Baumhueter S, Lai VK-M, Nunez L, Clarke BA (2001). Identification of ubiquitin ligases required for skeletal muscle atrophy. Science.

[b8] Boon H, Kostovski E, Pirkmajer S, Song M, Lubarski I, Iversen PO (2012). Influence of chronic and acute spinal cord injury on skeletal muscle Na^+^/K^+^-ATPase and phospholemman expression in humans. Am. J. Physiol. Endocrinol. Metab.

[b9] Brodie C, Sampson SR (1990). Veratridine-induced oscillations in membrane potential of cultured rat skeletal muscle: role of the Na-K pump. Cell. Mol. Neurobiol.

[b10] Clausen T (2003). Na^+^-K^+^ pump regulation and skeletal muscle contractility. Physiol. Rev.

[b11] Clausen T (2013). Quantification of Na^+^, K^+^ pumps and their transport rate in skeletal muscle: functional significance. J. Gen. Physiol.

[b12] Clausen T, Everts M (1991). K (+)-induced inhibition of contractile force in rat skeletal muscle: role of active Na (+)-K+ transport. Am. J. Physiol. Cell Physiol.

[b13] Conn JM, Annest JL, Gilchrist J (2003). Sports and recreation related injury episodes in the US population, 1997–99. Inj. Prev.

[b14] Desaphy J-F, Pierno S, Léoty C, George AL, De Luca A, Camerino DC (2001). Skeletal muscle disuse induces fibre type-dependent enhancement of Na+ channel expression. Brain.

[b15] Ditor DS, Hamilton S, Tarnopolsky MA, Green HJ, Craven BC, Parise G (2004). Na^+^, K^+^-ATPase concentration and fiber type distribution after spinal cord injury. Muscle Nerve.

[b16] Frank CB, Jackson DW (1997). Current concepts review-the science of reconstruction of the anterior cruciate ligament. J. Bone Joint Surg.

[b17] Frobell RB, Roos EM, Roos HP, Ranstam J, Lohmander LS (2010). A randomized trial of treatment for acute anterior cruciate ligament tears. N. Engl. J. Med.

[b18] Green HJ, Chin ER, Ball-Burnett M, Ranney D (1993). Increases in human skeletal muscle Na^+^-K^+^-ATPase concentration with short-term training. Am. J. Physiol. Cell Physiol.

[b19] Green HJ, Dahly A, Shoemaker K, Goreham C, Bombardier E, Ball-Burnett M (1999). Serial effects of high-resistance and prolonged endurance training on Na^+^-K^+^ pump concentration and enzymatic activities in human vastus lateralis. Acta Physiol. Scand.

[b20] Hackney KJ, Ploutz-Snyder LL (2011). Unilateral lower limb suspension: integrative physiological knowledge from the past 20 years (1991–2011). Eur. J. Appl. Physiol.

[b21] Hallal PC, Victora CG (2004). Reliability and validity of the international physical activity questionnaire (IPAQ). Med. Sci. Sports Exerc.

[b22] Hambly K, Griva K (2010). IKDC or KOOS which one captures symptoms and disabilities most important to patients who have undergone initial anterior cruciate ligament reconstruction?. Am. J. Sports Med.

[b23] Hansen O (2001). The *α*_1_ isoform of Na^+^, K^+^-ATPase in rat soleus and extensor digitorum longus. Acta Physiol. Scand.

[b24] Heir S, Nerhus TK, Røtterud JH, Løken S, Ekeland A, Engebretsen L (2010). Focal cartilage defects in the knee impair quality of life as much as severe osteoarthritis a comparison of knee injury and osteoarthritis outcome Score in 4 patient categories scheduled for knee surgery. Am. J. Sports Med.

[b25] Hopkins WG (2007). A spreadsheet to compare groups. Sportscience.

[b26] Irrgang JJ, Anderson AF, Boland AL, Harner CD, Kurosaka M, Neyret P (2001). Development and validation of the international knee documentation committee subjective knee form. Am. J. Sports Med.

[b27] Keays SL, Bullock-Saxton J, Keays AC, Newcombe P (2001). Muscle strength and function before and after anterior cruciate ligament reconstruction using semitendonosus and gracilis. Knee.

[b28] Knapik JJ, Staab JS, Harman EA (1996). Validity of an anthropometric estimate of thigh muscle cross-sectional area. Med. Sci. Sports Exerc.

[b29] Lee H-M, Cheng C-K, Liau J-J (2009). Correlation between proprioception, muscle strength, knee laxity, and dynamic standing balance in patients with chronic anterior cruciate ligament deficiency. Knee.

[b30] Leivseth G, Reikerås O (1994). Changes in muscle fiber cross-sectional area and concentrations of Na, K-ATPase in deltoid muscle in patients with impingement syndrome of the shoulder. J. Orthop. Sports Phys. Ther.

[b31] Levinger I, Levinger P, Trenerry MK, Feller JA, Bartlett JR, Bergman N (2011). Increased inflammatory cytokine expression in the vastus lateralis of patients with knee osteoarthritis. Arthritis Rheum.

[b32] Lorentzon R, Elmqvist L-G, Sjostrom M, Fagerlund M, Fugl-Meyer AR (1989). Thigh musculature in relation to chronic anterior cruciate ligament tear: muscle size, morphology, and mechanical output before reconstruction. Am. J. Sports Med.

[b33] MacDonald PB, Hedden D, Pacin O, Sutherland K (1996). Proprioception in anterior cruciate ligament-deficient and reconstructed knees. Am. J. Sports Med.

[b34] Madhavan S, Shields RK (2011). Neuromuscular responses in individuals with anterior cruciate ligament repair. Clin. Neurophysiol.

[b35] Makihara Y, Nishino A, Fukubayashi T, Kanamori A (2006). Decrease of knee flexion torque in patients with ACL reconstruction: combined analysis of the architecture and function of the knee flexor muscles. Knee Surg. Sports Traumatol. Arthrosc.

[b36] McKenna MJ, Schmidt TA, Hargreaves M, Cameron L, Skinner SL, Kjeldsen K (1993). Sprint training increases human skeletal muscle Na^+^-K^+^-ATPase concentration and improves K^+^ regulation. J. Appl. Physiol.

[b37] McKenna MJ, Bangsbo J, Renaud JM (2008). Muscle K^+^, Na^+^, and Cl disturbances and Na^+^-K^+^ pump inactivation: implications for fatigue. J. Appl. Physiol.

[b38] Medbø JI, Jebens E, Vikne H, Refsnes PE, Gramvik P (2001). Effect of strenuous strength training on the Na-K pump concentration in skeletal muscle of well-trained men. Eur. J. Appl. Physiol.

[b39] Miyasaka KC, Daniel DM, Stone ML, Hirshman P (1991). The incidence of knee ligament injuries in the general population. Am. J. Knee Surg.

[b40] Mizner RL, Petterson SC, Stevens JE, Vandenborne K, Snyder-Mackler L (2005). Early quadriceps strength loss after total knee arthroplasty: the contributions of muscle atrophy and failure of voluntary muscle activation. J. Bone Joint Surg.

[b41] Murphy KT, Snow RJ, Petersen AC, Murphy RM, Mollica J, Lee JS (2004). Intense exercise up-regulates Na^+^, K^+^-ATPase isoform mRNA, but not protein expression in human skeletal muscle. J. Physiol.

[b42] Narici MV, de Boer MD (2011). Disuse of the musculo-skeletal system in space and on earth. Eur. J. Appl. Physiol.

[b43] Nørgaard A, Kjeldsen K, Clausen T (1984). A method for the determination of the total number of 3 H-ouabain binding sites in biopsies of human skeletal muscle. Scand. J. Clin. Lab. Invest.

[b44] Perry BD, Levinger P, Serpiello FR, Caldow MK, Cameron-Smith D, Bartlett JR (2013). The effects of osteoarthritis and age on skeletal muscle strength, Na^+^, K^+^-ATPase content, gene and isoform expression. J. Appl. Physiol.

[b45] Petersen AC, Murphy KT, Snow RJ, Leppik JA, Aughey RJ, Garnham AP (2005). Depressed Na^+^-K^+^-ATPase activity in skeletal muscle at fatigue is correlated with increased Na^+^-K^+^-ATPase mRNA expression following intense exercise. Am. J. Physiol. Regul. Integr. Comp. Physiol.

[b46] Prodromos CC, Han Y, Rogowski J, Joyce B, Shi K (2007). A meta-analysis of the incidence of anterior cruciate ligament tears as a function of gender, sport, and a knee injury–reduction regimen. Arthroscopy.

[b47] Qiu F, Cole MH, Davids KW, Hennig EM, Silburn PA, Netscher H (2012). Enhanced somatosensory information decreases postural sway in older people. Gait Posture.

[b48] Radzyukevich TL, Neumann JC, Rindler TN, Oshiro N, Goldhamer DJ, Lingrel JB (2013). Tissue-specific Role of the Na, K-ATPase *α*_2_ Isozyme in Skeletal Muscle. J. Biol. Chem.

[b49] Roberts D, Ageberg E, Andersson G, Friden T (2007). Clinical measurements of proprioception, muscle strength and laxity in relation to function in the ACL-injured knee. Knee Surg. Sports Traumatol. Arthrosc.

[b50] Shields RK (2002). Muscular, skeletal, and neural adaptations following spinal cord injury. J. Orthop. Sports Phys. Ther.

[b51] Suomi R, Koceja DM (2000). Postural sway characteristics in women with lower extremity arthritis before and after an aquatic exercise intervention. Arch. Phys. Med. Rehabil.

[b52] Thomassen M, Christensen PM, Gunnarsson TP, Nybo L, Bangsbo J (2010). Effect of 2-wk intensified training and inactivity on muscle Na^+^-K^+^ pump expression, phospholemman (FXYD1) phosphorylation, and performance in soccer players. J. Appl. Physiol.

[b53] Valderrabano V, Von Tscharner V, Nigg BM, Hintermann B, Goepfert B, Fung TS (2006). Lower leg muscle atrophy in ankle osteoarthritis. J. Orthop. Res.

[b54] Wall BT, Dirks ML, Snijders T, Senden JM, Dolmans J, Loon L (2014). Substantial skeletal muscle loss occurs during only 5 days of disuse. Acta Physiol.

[b55] Welinder C, Ekblad L (2011). Coomassie staining as loading control in Western blot analysis. J. Proteome Res.

[b56] Willems T, Witvrouw E, Verstuyft J, Vaes P, De Clercq D (2002). Proprioception and muscle strength in subjects with a history of ankle sprains and chronic instability. J. Athl. Train.

[b57] Williams GN, Buchanan TS, Barrance PJ, Axe MJ, Snyder-Mackler L (2005a). Quadriceps weakness, atrophy, and activation failure in predicted noncopers after anterior cruciate ligament injury. Am. J. Sports Med.

[b58] Williams GN, Snyder-Mackler L, Barrance PJ, Buchanan TS (2005b). Quadriceps femoris muscle morphology and function after ACL injury: a differential response in copers versus non-copers. J. Biomech.

